# Supplementary material for: Probing the Effect of Acidosis on Tether-Mode Mechanotransduction of Proprioceptors
Source: Int J Mol Sci. 2023 Aug 14;24(16):12783. doi: 10.3390/ijms241612783 (PMC10454156; doi:10.3390/ijms241612783)
Supplement: Supplementary file 1 [file ijms-24-12783-s001.zip › ijms-2518627-supplementary.pdf]

International Journal of
Molecular Sciences

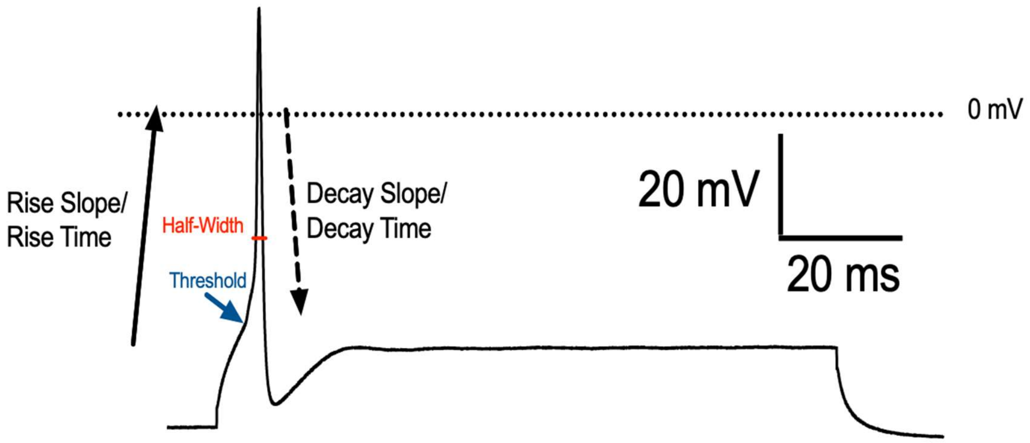

Supplement Figure S1. The three AP firing patterns were recorded in Pv+ neurons via whole-cell patch clamping. The analysis parameters of an action potential were analyzed using Clampfit 11.2 (Molecular Devices). The DRG neuron was held at -70 mV. The software measured the rise slope/rise time from 10% to 90 % of peak amplitude, decay slope/decay time from 90% to 15% of peak amplitude, half-width, and threshold.

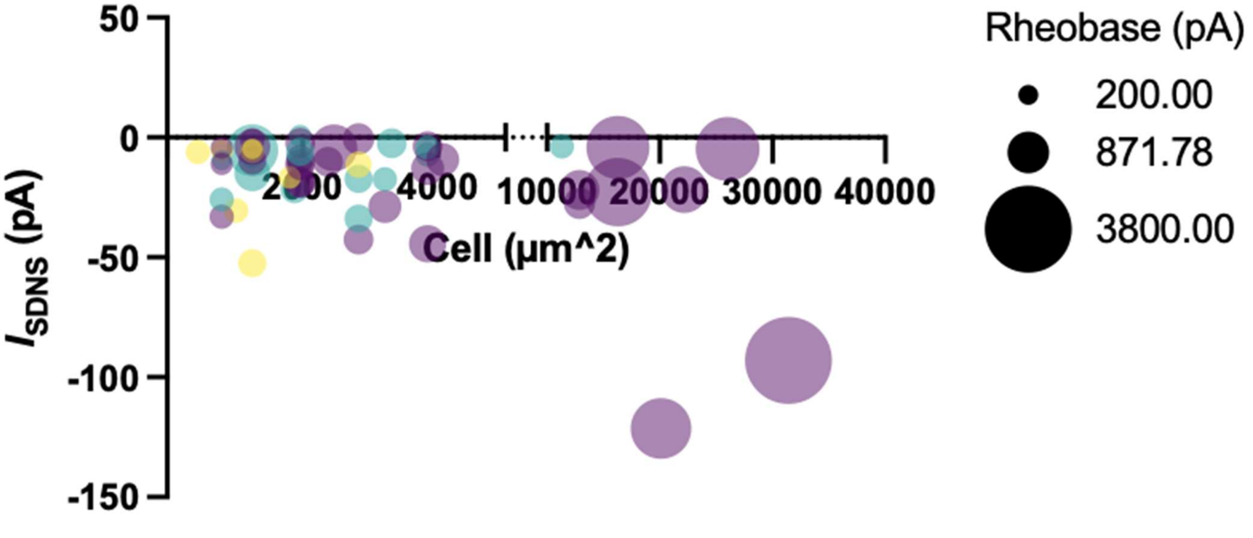

Supplement Figure S2. Diagram of an indentation current at pH 7.4, cell size, rheobase, and AP firing profile. The x-axis shows the I_{SDNS} at pH 7.4, and the y-axis shows the cell size. The size of the dots indicates the range of the rheobase, and the single, burst, and tonic APs are, respectively, colored in yellow, green, and blue (*n* = 63).

Supplement Table S1. The proportion of firing patterns, SDNS responders, and acidosis responders in Pv+ DRG neurons.

**SDNS (pH 7.4)**			**APETx2 (SDNS)**			pH 6.8			APETx2 (pH 6.8)			Firing Pattern		
**Response**	**N**	**Percentage**	**Response**	**N**	**Percentage**	Response	N	Percentage	Response	N	Percentage	Types	N	Percentage
Sensitive	25	59.52%	Sensitive	23	92.00%	w/	19	82.61%	Sensitive (Group 1)	11	57.89%	single	5	45.45%
												Burst	3	27.27%
												Tonic	3	27.27%
									Insensitive (Group 2)	8	42.11%	single	5	62.50%
												Burst	2	25.00%
												Tonic	1	12.50%
						w/o (Group 3)	4	17.39%				single	3	75.00%
												Burst	1	25.00%
												Tonic	0	0.00%
			Insensitive	2	8.00%	w/	2	100.00%	Sensitive (Group 4)	2	100.00%	single	2	100.00%
												Burst	0	0.00%
												Tonic	0	100.00%
									Insensitive	0	0.00%	single	0	NA
												Burst	0	NA
												Tonic	0	NA
						w/o	0	0.00%				single	0	NA
												Burst	0	NA
												Tonic	0	NA
Insensitive	17	40.48%				w/	11	64.71%	Sensitive (Group 5)	6	54.55%	single	3	50.00%
												Burst	2	33.33%
												Tonic	1	16.67%
									Insensitive (Group 6)	5	45.45%	single	2	40.00%
												Burst	3	60.00%
												Tonic	0	0.00%
						w/o	6	35.29% (Group 7)				single	5	83.33%
												Burst	1	16.67%
												Tonic	0	0.00%
